# Atorvastatin mitigates memory deficits and brain monocyte infiltration in chronic hypercholesterolemia

**DOI:** 10.18632/aging.205217

**Published:** 2023-11-17

**Authors:** Fengchao Gong, Qian Shi, Xiaojie Mou, Kang Wang, Qianqian Wang, Haitao Wang

**Affiliations:** 1 Department of Neurology, The Second Affiliated Hospital of Shandong First Medical University, Tai'an 271000, Shandong, China; 2 Department of Ultrasound, The Second Affiliated Hospital of Shandong First Medical University, Tai'an 271000, Shandong, China

**Keywords:** cognitive impairment, chronic hypercholesterolemia, Atorvastatin, monocyte infiltration

## Abstract

Mild cognitive impairment (MCI) is a common symptom observed in people over 60 years old and is found to be aggravated by hypercholesterolemia. Severe neuroinflammation induced by BBB dysfunction and monocyte infiltration might be responsible for neuron damage and cognitive impairment. Atorvastatin is a lipid-lowering drug that is widely applied for the treatment of cardiovascular diseases. However, the potential function of Atorvastatin in hypercholesterolemia-induced MCI remains uncertain. Our research will explore the potential therapeutic function of Atorvastatin in memory deficits induced by chronic hypercholesterolemia. ApoE^-/-^ mice were utilized to mimic the state of chronic hypercholesterolemia and were divided into four groups. Animals in the WT and ApoE^-/-^groups were orally administered with normal saline, while WT mice in the Atorvastatin group and ApoE^-/-^ mice in the ApoE^-/-^+ Atorvastatin group were orally administered with 10 mg/kg/day Atorvastatin. Markedly increased plasma cholesterol levels reduced RI in the long-term memory test and the spatial short-term memory test, declined mobility in the open field test, and downregulated PSD-95 and BDNF were observed in ApoE^-/-^ mice, all of which were signally reversed by Atorvastatin. Moreover, the percentages of brain Ly6C^hi^ CD45^+^ cells and CD3^+^ CD45^+^ cells, as well as the blood Ly6C^hi^ CD45^+^ cells, plasma IL-12/IL-23 levels and IL-17 level were found notably increased in ApoE^-/-^ mice, all of which were largely repressed by Atorvastatin. Lastly, the increased BBB permeability, decreased ZO-1 and occludin levels, and reduced KLF2 level were markedly abolished by Atorvastatin. Collectively, Atorvastatin mitigated memory deficits and brain monocyte infiltration in ApoE^-/-^ mice.

## INTRODUCTION

Mild cognitive impairment (MCI) refers to the clinical phenomenon of mild memory impairment or certain cognitive impairment in people over 60 years old (including 60 years old), but there is no sufficient evidence to confirm it as dementia [1]. Studies have shown that multiple risk factors contribute to the development of MCI, including hypertension, hypercholesterolemia, diabetes and atherosclerosis, which aggravate MCI and results in dementia [2, 3]. Cholesterol metabolism in brain tissue is found to be the core of the pathophysiology of cognitive disorders, and the level of cholesterol is a potential risk factor for the occurrence of MCI [4]. Zou Y et al. [5] observed 597 elderly patients over 60 years old and found that total cholesterol was an independent risk factor for MCI, and cardiovascular diseases increased the risk of MCI. Toro P et al. [6] conducted a 14-year follow-up of a birth cohort and found that those with high serum total cholesterol at baseline showed a higher risk of developing MCI later in life. There are two main sources of cholesterol in brain tissue: one is from astrocyte synthesis, by which cholesterol is transported to neurons via ApoE -dependent transporters, while the second one is that the low-density lipoprotein of the circulatory system enters brain tissue through the blood-cerebrospinal fluid barrier via ApoE-dependent receptors [7]. Pathological changes to vascular endothelial cells are induced by hypercholesterolemia, mainly manifested in structural changes and dysfunction, resulting in impaired perfusion of brain tissue, obstruction of normal transportation of oxygen, glucose and other substances, and inducing the occurrence of cerebrovascular terminal events [8]. On the one hand, the pathological changes of atherosclerosis are accelerated and on the other hand, the speed of cerebral blood flow is slowed down and brain metabolism is abnormal, thus accelerating the development of cognitive dysfunction [9]. In addition, the increase in serum cholesterol levels directly induces pathophysiological changes and death of neurons. The metabolism of amyloid precursor protein (APP) by nerve cells is affected by the increase in serum cholesterol levels, inducing the abnormal secretion of Aβ and its deposition in brain tissue, eventually triggering the degeneration and death of neurons to induce cognitive dysfunction [10]. Aβ disrupts lipid metabolism and mitochondria function in cardiac cells [11]. It was also reported that senile plaques arise from Aβ- and Cathepsin D-enriched mixtures leaking out during intravascular haemolysis and microaneurysm rupture [12]. Controlling the cholesterol level is of great significance for the prevention of MCI.

Atorvastatin, a lipid-lowering drug, is widely used in the prevention and treatment of cardiovascular diseases. Atorvastatin is found to greatly reduce the mortality rate of heart failure and shows a great effect on other cardiovascular diseases. Studies have reported that, in addition to lipid-lowering effects, Atorvastatin is reported to inhibit vasoconstriction, improve endothelial function, reduce pulmonary vasodilatation, inhibit the release of inflammatory factors, and reduce the levels of inflammatory markers such as TNF-α and CRP [13]. In the clinic, Atorvastatin is evidenced to improve endothelial function in ischemic heart failure patients via regulating the mobilization of endothelial progenitor cells [14]. Furthermore, the protective property of Atorvastatin against endothelial cell damage was observed [15]. Recently, the neuroprotective effect of Atorvastatin has also been studied. Taniguti et al. reported that Atorvastatin prevented LPS-induced depressive-like behavior by reducing the secretion of TNF-α and impressing oxidative stress [16]. A randomized clinical trial on Atorvastatin treating chronic subdural hematoma (CSDH) was conducted. They found that 45.9% of the total patients taking Atorvastatin (20 mg daily) had significantly improved neurological function [17]. Interestingly, Gülay Üzüm and colleagues reported that the administration of Atorvastatin displayed a protective effect on memory deficit and seizure susceptibility in Pentylenetetrazole-kindled rats [18].

Our study supposed that Atorvastatin may be beneficial for memory deficits and brain monocyte infiltration in chronic hypercholesterolemia. So, our research will examine the potential therapeutic function of Atorvastatin in memory deficits induced by chronic hypercholesterolemia and intends to explore more approaches for the treatment of chronic hypercholesterolemia-related diseases.

## MATERIALS AND METHODS

### Animals and grouping

ApoE^-/-^ aged mice were applied in our study to mimic the state of chronic hypercholesterolemia. 10 Normocholesterolemic WT and 30 ApoE^-/-^ aged male mice at the age of 16 months were obtained from Charles River (Beijing, China). Four groups were divided: WT, Atorvastatin, ApoE^-/-^, and ApoE^-/-^+ Atorvastatin. Animals in the WT and ApoE^-/-^groups received oral administration of normal saline, while WT mice in the Atorvastatin group and ApoE^-/-^ mice in the ApoE^-/-^+ Atorvastatin group received oral administration of 10 mg/kg/day Atorvastatin (Cat#C106840, Cuiechem, Shanghai, China) for 7 days [19].

### Detection of plasma cholesterol level

The plasma cholesterol level in each animal was detected using an automatic biochemical analyzer (Beckman, USA). For internal quality control, commercial control sera (Randox, Crumlin, UK) were used.

### Open field test

A camera was placed in the center of the open field test chamber (25 cm×25 cm×38 cm), and the chamber was hollow. Mice were placed in the center of the test chamber and allowed to move freely for 5 min. The mobility (%) of mice in the specified time was recorded, which reflected the spontaneous activity ability of mice.

### Novel object recognition (NOR)

The experiment was conducted in an open box at the size of 25 cm × 25 cm × 40 cm. The test is mainly divided into three stages: adaptation, familiarity, and recognition. On the first day of adaptation, mice were placed at one end of the test box with their back to the operator and allowed to move freely for 10 minutes to adapt to the environment. On the second day of familiarity, two identical identified objects were placed in the test box and mice were allowed to move freely for 10 minutes. Then, the recognition time of the two objects was recorded, which was the time when the mouse’s mouth came within 2 cm of the object and/or touched the object with its nose. The bottom of the box and the object were cleaned with 75% ethanol for each mouse after the experiment. On the third day of the test, one of the objects was replaced with a new object of different color and shape. Mice were exposed to old objects for 10 min. 5 min or 24 hours later, the mouse was exposed to new objects for 10 min. The recognition time of new and old objects within 10 min was recorded, and RI was calculated. RI=new object recognition time/(new object recognition time + old object recognition time). The short-term memory was tested by a delay interval of 5 min, while a long-term memory was tested by a delay interval of 24 hours [20].

### RT-PCR

After the mice were sacrificed, bilateral hippocampi were quickly separated and placed in a glass homogenizer. Total mRNA was extracted according to Trizol reagent instructions. cDNA synthesis was performed by RT-PCR reverse transcription kit (MedChemExpress, USA) operation. PCR amplification mixed system (Lifeint, Xiamen, China) was conducted on the ABI7500 Real-Time PCR (Thermo Fisher Scientific, USA). The internal reference gene was GAPDH and the gene level was determined utilizing the 2^−ΔΔCt^ method. The primer sequences were listed in Table 1.

**Table 1:** Primer sequences.

	**Forward (5’-3’)**	**Reverse (5’-3’)**
PSD-95	GCTCCAAAGCCCCATCATT	AAGGCAGGCTGTCCGTGTCCGTATCC
BDNF	CAGTGGCTGGCTCTCATACC	CGGAAACAGAACGAACAGAA
ZO-1	TATTATGGCACATCAGCACG	TGGGCAAACAGACCAAGC
Occludin	GAGGGTACACAGACCCCAGA	CAGGATTGCGCTGACTATGA
KLF2	GCACGGATGAGGACCTAAAC	GTAGCTGCAAGTATGTGTGG
GAPDH	CCTGTGGCATCCATGAAACTAC	AGCTAGGAGCCAGGGCAGTAA

### Flow cytometry

After the mice were sacrificed, the brain tissue and blood were collected and the single-cell suspension was achieved according to the dosage recommended in the antibody instructions. 0.5 μL FITC anti-mouse Ly6C, 0.5 μL PE anti-mouse CD45, 0.5 μL PerCP/Cy5.5 anti-mouse CD3 antibody, 8.6 μL cell staining buffer were added into cells and incubated at room temperature away from light for 15 min, followed by loading to the flow cytometry (BD, USA) for detection. The percentage of brain Ly6C^hi^ cells (%CD45^+^ viable cells) and the percentage of brain CD3^+^ cells (%CD45^+^ viable cells) were recorded.

### Diffusion of sodium fluorescein assay

Mice were injected with 0.1 mL 10% NaFI and then intraperitoneally administered with 4% chloral hydrate for 45 min, followed by opening the chest cavity and intubating the cannula into the aorta through the left ventricle. After cutting the right atrium to perfuse the normal saline until the clear fluid flowed out of the right atrium, the brain was taken. Then, 0.6 mL PBS was added to the brain tissue, and the homogenization of brain tissue was made as the test sample. Then, samples were centrifugated at 3000 r/min for 5 min and the supernatant was diluted with 20% TCA (1:10), followed by incubation at 4° C for 24 h. Samples were then centrifuged at 10 000 r/min for 15 min to collect the supernatant, which was then diluted with the same volume of Tris-HCL buffer. Fluorescence intensity (490/514 nm) was measured and the BBB permeability was determined by the amount of NaFI per milligram of brain tissue protein.

### ELISA assay for cytokine detection

The secretion of IL-12/IL-23 and IL-17 is detected with Commercial ELISA kits (Cat#EK0508, EK5178, Signalway Antibody, Nanjing, China). The plasma of each animal was collected and diluted at a 1:1 ratio, which was loaded into wells and introduced with 50 μL biotin-labeled antibody IL-12/IL-23 and IL-17. Following 60 min incubation at 37° C, the solution was cleared and 80 μL HRP-loaded secondary antibody was added, followed by half an hour culture at 37° C. Subsequently, 50 μL TMB substrates were introduced and cultured at 37° C for 10 min, followed by loading 50 μL stop solution and detecting the OD value utilizing a microplate reader (MD, USA).

### Western blotting assay

Brain tissues were collected for the extraction of total proteins, which were quantified with the BCA method, followed by conducting the separation with the 12% SDS-PAGE. After transferring proteins to the PVDF membrane, 5% skim milk was applied for blocking. The primary antibodies against ZO-1 (1:2000, Cat#sc-8146, Santa Cruz Biotechnology, USA), occludin (1:2000, Cat#ab31721, Abcam, UK), KLF2 (1:1000, Cat#ab194486, Abcam, UK), and β-actin (1:5000, Bosterbio, USA) were added, followed by incubation with the secondary antibody (1:1000, Bosterbio, USA). After 60 min incubation, the ECL solution was added for exposure in a film-developing equipment (OPTIMAX, Sunjune, China). The results were scanned and target bands were selected. Then the sum optical density of the bands was then quantified using the software Image J (NIH, USA) and exported for statistical analysis.

### Statistical analysis

Data were expressed as mean±standard deviation (S.D.). Statistics were performed using the software SPSS version 24. The comparison was analyzed using a one-way analysis of variance (ANOVA) method with the post-hoc Scheffe test. P<0.05 was taken as a statistically significant difference.

### Data availability

The data will be made available on request.

## RESULTS

### Atorvastatin decreased the plasma cholesterol levels in aged ApoE^-/-^ mice

**Figure 1:**
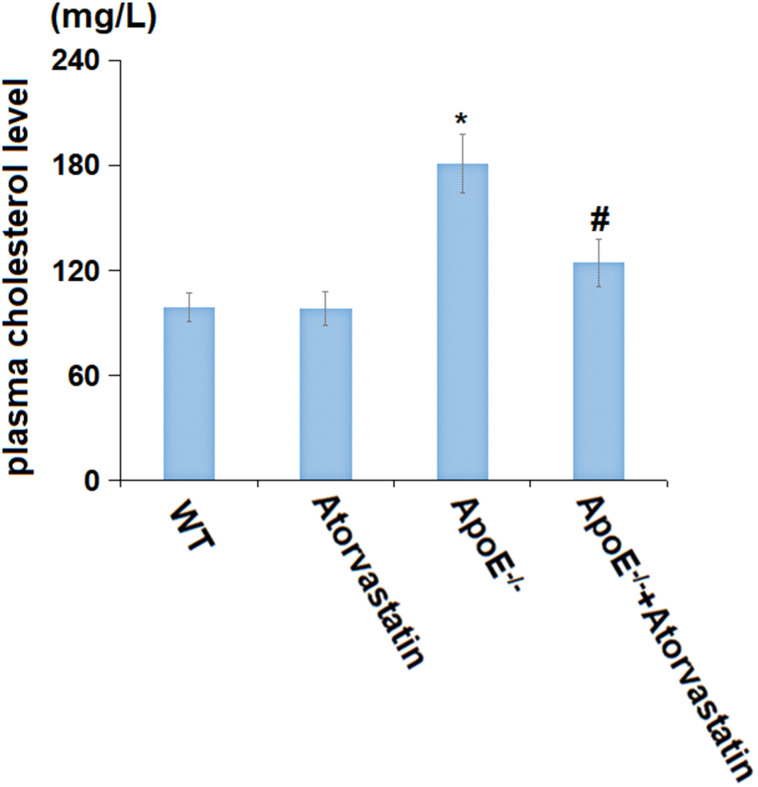
**Atorvastatin decreased the plasma cholesterol level in aged ApoE^-/-^ mice.** The plasma cholesterol level was tested by an automatic biochemical analyzer (mg/L) (*, P<0.05 vs. vehicle; #, ##, P<0.01, 0.05 vs. ApoE^-/-^ mice group).

Firstly, the plasma cholesterol levels of each animal were checked. The average plasma cholesterol levels in the WT and Atorvastatin groups were 99.2 mg/L and 98.4 mg/L, respectively, which were signally increased to 180.9 mg/L in ApoE^-/-^ mice. After dosing with Atorvastatin, the plasma cholesterol levels in ApoE^-/-^ mice were markedly repressed to 124.7 mg/L (Figure 1). A repressive effect of Atorvastatin against high plasma cholesterol levels in ApoE^-/-^ mice was observed.

### Treatment with Atorvastatin alleviated memory impairment in aged ApoE^-/-^ mice spatial memory

**Figure 2:**
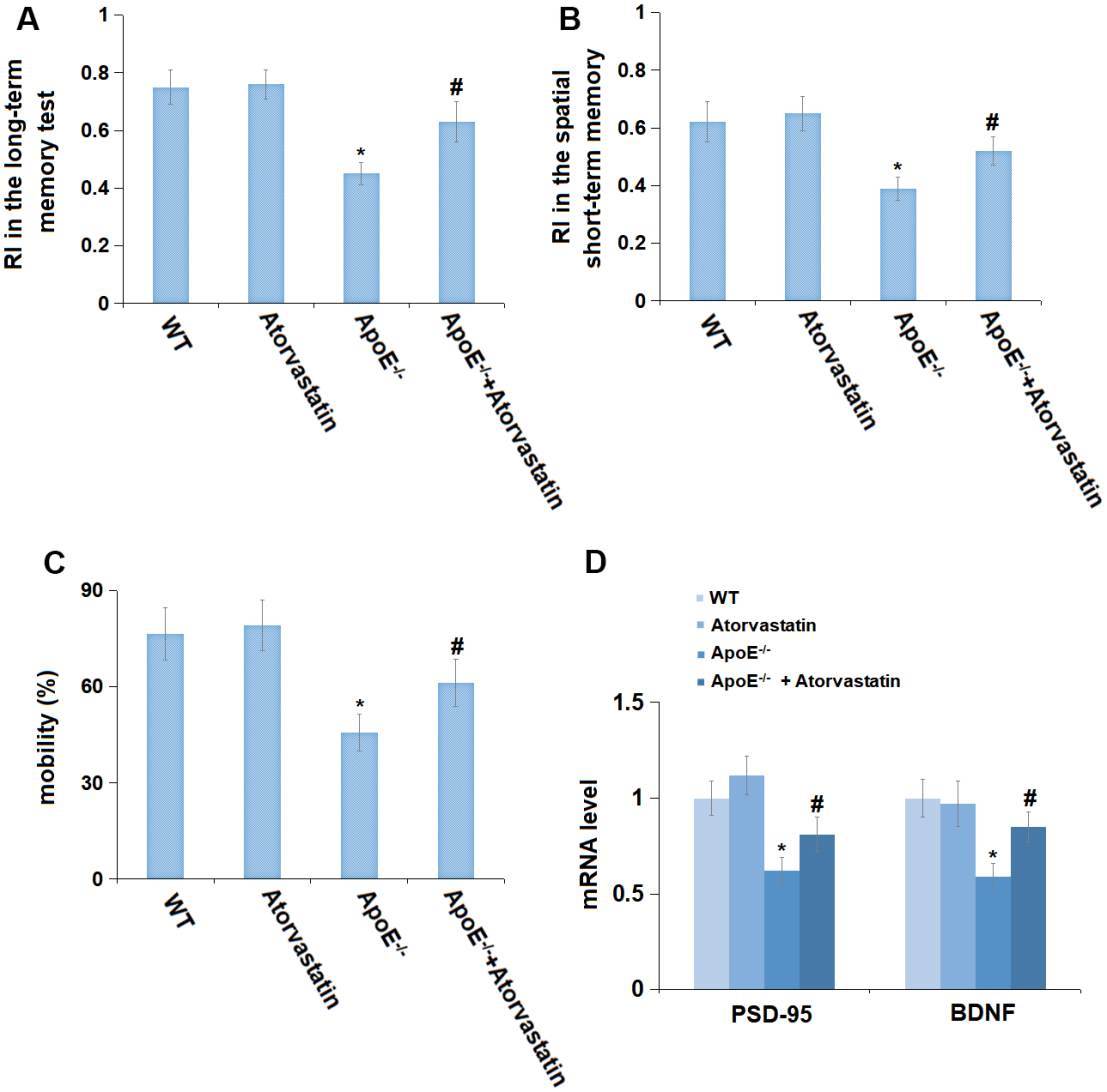
**Treatment with Atorvastatin alleviated memory impairment in aged ApoE^-/-^ mice.** (**A**) Recognition index (RI) in the long-term memory test; (**B**) Recognition index (RI) in the spatial short-term memory test; (**C**) The mobility (%) was measured in the open field test. (**D**) The mRNA level of *PSD-95* and *BDNF* in the hippocampus tissue was checked by RT-PCR (*, P<0.05 vs. vehicle; #, P<0.05 vs. ApoE^-/-^ mice group).

To test the degree of memory impairment in mice, the open field test and NOR were conducted. RI in the long-term memory test in the WT and Atorvastatin groups was 0.75 and 0.76, respectively, and was notably reduced to 0.45 in ApoE^-/-^ mice, which was then markedly increased to 0.63 by Atorvastatin (Figure 2A). Moreover, the RI in the spatial short-term memory test in the WT, Atorvastatin, ApoE^-/-^, and ApoE^-/-^+ Atorvastatin groups was 0.62, 0.65, 0.39, and 0.52, respectively (Figure 2B). In the open field test, the mobility in the WT and Atorvastatin groups was 76.5% and 79.2% and was observably decreased to 45.7% in ApoE^-/-^ mice, which was markedly elevated to 61.2% by Atorvastatin (Figure 2C). Furthermore, the mRNA levels of PSD-95 and BDNF were minorly changed in the Atorvastatin group but notably downregulated in ApoE^-/-^ mice, which was markedly reversed by Atorvastatin (Figure 2D). Memory impairment in aged ApoE^-/-^ mice was found significantly ameliorated by Atorvastatin.

### Atorvastatin repressed the monocyte infiltration in aged ApoE^-/-^ mice

**Figure 3:**
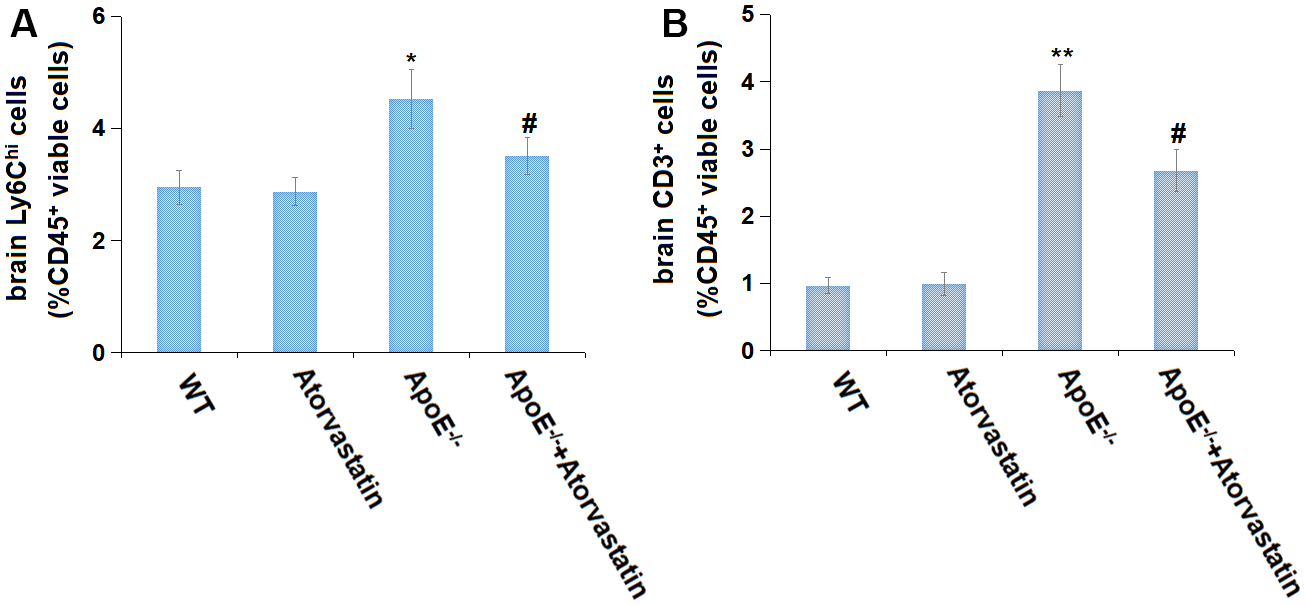
**Atorvastatin repressed the monocyte infiltration in aged ApoE^-/-^ mice.** (**A**) The percentage of brain Ly6C^hi^ cells (%CD45^+^ viable cells) was detected by flow cytometry; (**B**) The percentage of brain CD3^+^ cells (%CD45^+^ viable cells) was detected by flow cytometry (*, **, P<0.05, 0.01 vs. vehicle; #, P<0.05 vs. ApoE^-/-^ mice group).

The infiltration of monocytes was found to be an inducer of MCI in chronic hypercholesterolemia ^20^. The percentages of brain Ly6C^hi^ cells (%CD45^+^ viable cells) in the WT, Atorvastatin, ApoE^-/-^, and ApoE^-/-^+ Atorvastatin groups were 2.95%, 2.87%, 4.53%, and 3.52%, respectively (Figure 3A). Furthermore, the percentage of brain CD3^+^ cells (%CD45^+^ viable cells) in the WT and Atorvastatin groups was maintained around 1% but was markedly increased to 3.87% in ApoE^-/-^ mice, then reduced to 2.68% by Atorvastatin (Figure 3B). A suppressive property of Atorvastatin against the monocyte infiltration in aged ApoE^-/-^ mice was observed.

### Atorvastatin inhibited the immune system activation in aged ApoE^-/-^ mice

**Figure 4:**
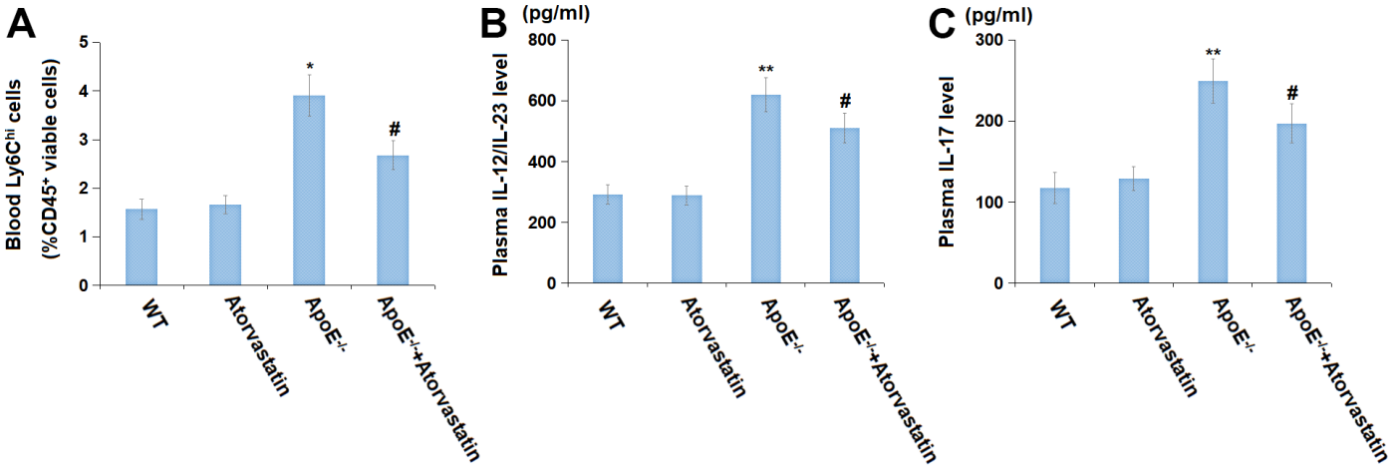
**Atorvastatin inhibited the immune system activation in aged ApoE^-/-^ mice.** (**A**) Blood Ly6C^hi^ cells (%CD45^+^ viable cells) was detected by flow cytometry; (**B**) Plasma IL-12/IL-23 level (pg/ml); (**C**) Plasma IL-17 level (pg/ml) (*, **, P<0.05, 0.01 vs. vehicle; #, P<0.05 vs. ApoE^-/-^ mice group).

Subsequently, the peripheral inflammation in each mouse was checked. The blood Ly6C^hi^ cells percentages (%CD45^+^ viable cells) in the WT, Atorvastatin, ApoE^-/-^, and ApoE^-/-^+ Atorvastatin groups were 1.57%, 1.66%, 3.91%, and 2.68%, respectively (Figure 4A). Moreover, the plasma IL-12/IL-23 level in the Atorvastatin group was slightly changed from 292.5 to 288.6 pg/mL but was markedly increased to 619.4 pg/mL in ApoE^-/-^ mice, which was then signally repressed to 510.6 pg/mL by Atorvastatin (Figure 4B). Furthermore, the plasma IL-17 levels in the WT, Atorvastatin, ApoE^-/-^, and ApoE^-/-^+ Atorvastatin groups were 117.4, 129.3, 249.5, and 197.3 pg/mL, respectively (Figure 4C). The immune system activation in aged ApoE^-/-^ mice was found markedly repressed by Atorvastatin.

### Atorvastatin prevented the increased BBB permeability in aged ApoE^-/-^ mice

**Figure 5:**
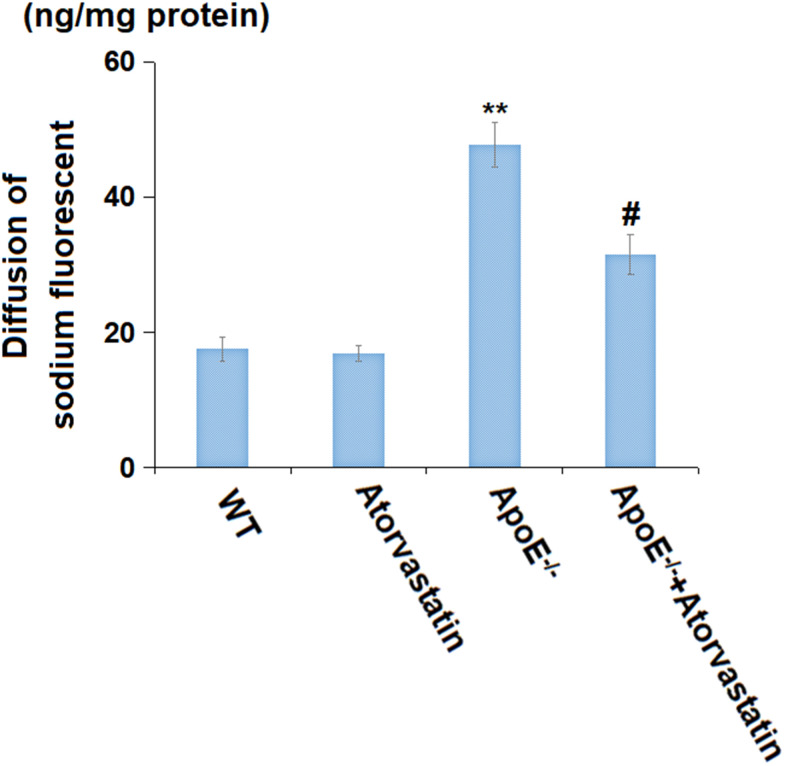
**Atorvastatin prevented the enlarged blood-brain barrier (BBB) permeability in aged ApoE^-/-^ mice.** Diffusion of sodium fluorescent in treated animals’ cortex (ng/mg protein) was used to index BBB integrity (**, P<0.01 vs. vehicle; #, P<0.05 vs. ApoE^-/-^ mice group).

Increased BBB permeability is one of the inducers for monocyte infiltration in the brain [21]. The diffusion of sodium fluorescent in the treated animals’ cortices in the Atorvastatin group was minorly altered from 17.6 to 16.9 ng/mg protein but was prominently promoted to 47.8 ng/mg protein in ApoE^-/-^ mice, which was then markedly reduced to 31.5 ng/mg protein by Atorvastatin (Figure 5), implying a suppressive effect of Atorvastatin on the increased BBB permeability in aged ApoE^-/-^ mice.

### Atorvastatin restored the expression of tight junction proteins in the cortices of aged ApoE^-/-^ mice

**Figure 6:**
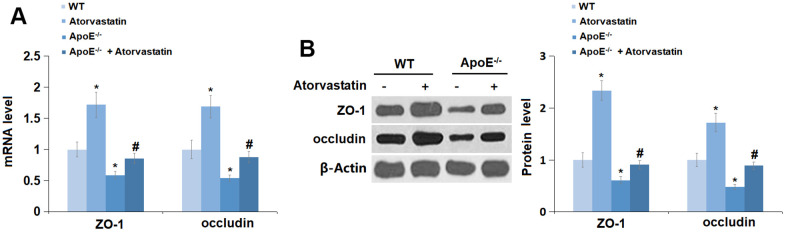
**Atorvastatin restored the expression of tight junction proteins in the cortex of aged ApoE^-/-^ mice.** (**A**) mRNA of ZO-1 and occludin was measured by RT-PCR; (**B**) Protein of ZO-1 and occludin was measured using immunostaining (*, P<0.05 vs. vehicle; #, P<0.05 vs. ApoE^-/-^ mice group).

Tight junctions are critical for the maintenance of BBB permeability [22]. The levels of ZO-1 and occludin were markedly increased in the Atorvastatin group but were notably decreased in ApoE^-/-^ mice, which was signally reversed by Atorvastatin (Figure 6A, 6B), suggesting that the declined levels of tight junction proteins in the cortices of aged ApoE^-/-^ mice were restored by Atorvastatin.

### Atorvastatin reversed the downregulation of KLF2 in the cortices of aged ApoE^-/-^ mice

**Figure 7:**
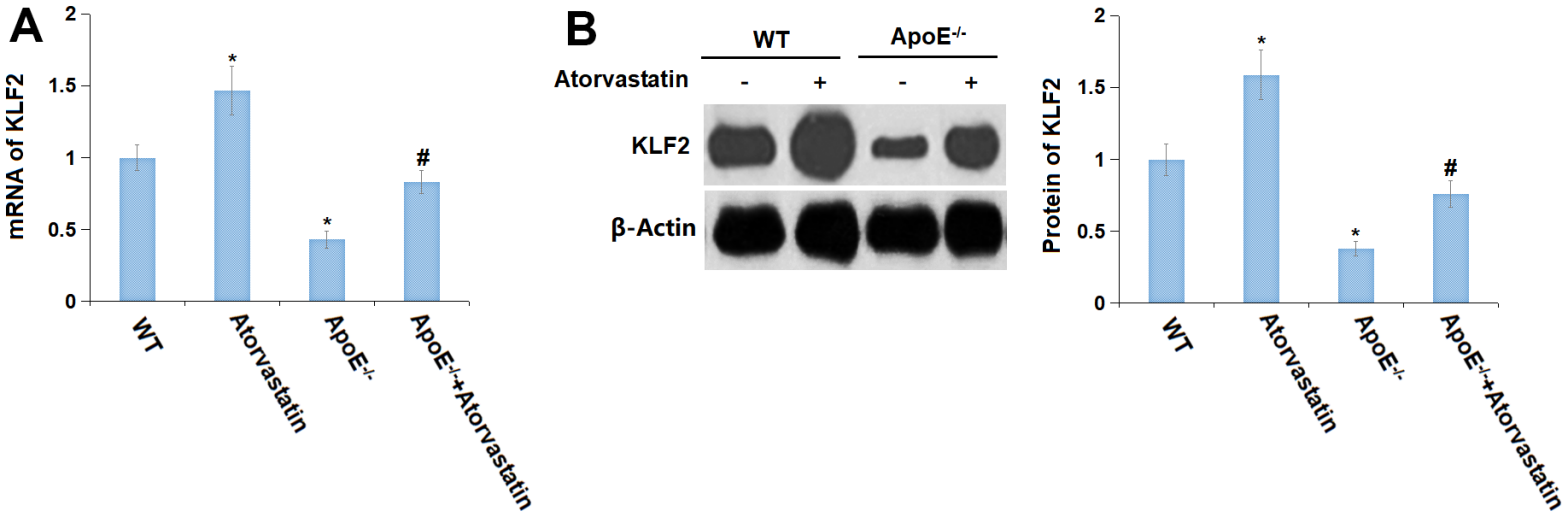
**Atorvastatin reversed the downregulation of KLF2 in the cortex of aged ApoE^-/-^ mice.** (**A**) mRNA of KLF2; (**B**) Protein of KLF2 (*, P<0.05 vs. vehicle; #, P<0.05 vs. ApoE^-/-^ mice group).

KLF2 is a transcriptional factor that is found to participate in regulating the expression of tight junction proteins [23]. In our research, KLF2 was found signally upregulated in the Atorvastatin group but markedly downregulated in ApoE^-/-^ mice, the level of which was then prominently elevated by Atorvastatin (Figure 7A, 7B).

## DISCUSSION

ApoE is widely distributed in the body, and ApoE in peripheral plasma is mainly synthesized by the liver, macrophages, bones, ovaries, and kidneys, while ApoE in the central nervous system is mainly synthesized by astrocytes [24]. ApoE is reported to participate in the body's lipid metabolism through a variety of pathways and is an important intrinsic factor affecting the body's blood lipid levels. The absence of ApoE leads to hypercholesterolemia [25]. In animal studies, ApoE^-/-^ mice are often used to simulate the pathological symptoms of hypercholesterolemia [26]. In the elderly population over 80 years old, higher levels of cholesterol are closely related to cognitive impairment [27]. In our research, ApoE^-/-^ mice were utilized to mimic the symptoms of hypercholesterolemia, and high plasma cholesterol levels were observed in the ApoE^-/-^ mice, in line with Tsukahara’s research [28]. Furthermore, consistent with the report presented by Nicholas [20], a markedly declined RI in both the long-term memory test and the spatial short-term memory test, as well as decreased mobility in the open field test, were observed in ApoE^-/-^ mice. Following the administration of Atorvastatin, the memory impairment was signally reversed, accompanied by a declined plasma cholesterol level, implying that Atorvastatin alleviated the hypercholesterolemia-induced memory deficits in mice. *PSD-95* and *BDNF* are two critical brain-located proteins responsible for cognition and memory [29, 30]. Signally reduced mRNA levels of *PSD-95* and *BDNF* were found in ApoE^-/-^ mice, which were markedly increased by Atorvastatin, further verifying the protective property of Atorvastatin against the memory impairment in hypercholesterolemia mice.

Neuroinflammation is a key factor leading to neurodegenerative diseases of the central nervous system [31, 32]. Evidence shows that peripheral inflammation is one of the main factors inducing neuroinflammation in the brain, and pro-inflammatory cytokines have been shown to cross the BBB and trigger the central inflammatory response. Damage to the BBB may exacerbate central inflammation by allowing peripheral immune cells to enter the brain and aggravate neuroinflammation [33]. Obesity is a key factor in peripheral systemic inflammation and a previous study has shown that excessive saturated fatty acids in obese patients are found to cross the BBB and directly affect the activation of inflammation in the brain. Furthermore, type 2 diabetes caused by obesity accelerates memory dysfunction and neuroinflammation in mouse models of Alzheimer's disease [34]. In our research, monocyte infiltration in brain tissue and enhanced peripheral inflammation were observed in aged ApoE^-/-^ mice, which were notably alleviated by Atorvastatin, implying that the protective function of Atorvastatin in ApoE^-/-^ mice might be correlated with the inhibition of inflammation. Moreover, the increased BBB permeability observed in aged ApoE^-/-^ mice was ameliorated by Atorvastatin, suggesting that Atorvastatin might suppress neuroinflammation by protecting against BBB disruption.

Tight junctions are protein molecular complex structures located at the top of brain microvascular endothelial cells in the BBB, and can be segmented into transmembrane proteins, cytoplasmic attachment proteins, and cytoskeletal proteins according to subcellular localization. Transmembrane proteins are mainly composed of occludin, claudins family, and junctional adhesion molecules (JAMs). Cytoplasmic attachment proteins include zonula occludens (ZO) proteins, afadin (AF-6), and calcium/calmodulin-dependent serine protein kinases, among which claudin-5, occludin, and ZO-1 are closely related to the impairment of BBB function [35–37]. In our research, tight junction proteins were found signally downregulated in aged ApoE^-/-^ mice, consistent with the declined level of ZO-1 in the model of atherosclerosis (ApoE^-/-^ mice) reported by Yan [38]. After the administration of Atorvastatin, the levels of tight junction proteins were recovered. KLF2 is a transcriptional factor that is reported to mediate the expression of tight junction proteins [39]. We found that the recovered expression of tight junction proteins was accompanied by an upregulation of KLF2, implying that the protective function of Atorvastatin against memory deficits in aged ApoE^-/-^ mice might be corrected with KLF2-mediated BBB repair. In future research, the potential mechanism will be verified by testing the protective function of Atorvastatin in KLF2-slienced brain microvascular endothelial cells. Additionally, the further mechanism will be examined in our future study.

Collectively, Atorvastatin might possess a promising effect in mitigating memory deficits and brain monocyte infiltration in ApoE^-/-^ mice.
